# Receptor and metabolic insights on the ability of caffeine to prevent alcohol-induced stimulation of mesolimbic dopamine transmission

**DOI:** 10.21203/rs.3.rs-4289552/v1

**Published:** 2024-06-11

**Authors:** Riccardo Maccioni, Valentina Bassareo, Giuseppe Talani, Simone Zuffa, Yasin El Abiead, Irene Lorrai, Tomoya Kawamura, Sofia Pantis, Roberta Puliga, Romina Vargiu, Daniele Lecca, Paolo Enrico, Alessandra Peana, Laura Dazzi, Pieter C. Dorrestein, Pietro Paolo Sanna, Enrico Sanna, Elio Acquas

**Affiliations:** The Scripps Research Institute; The Scripps Research Institute; The Scripps Research Institute; The Scripps Research Institute; The Scripps Research Institute; The Scripps Research Institute; The Scripps Research Institute; The Scripps Research Institute; The Scripps Research Institute; The Scripps Research Institute; The Scripps Research Institute; The Scripps Research Institute; The Scripps Research Institute; The Scripps Research Institute; The Scripps Research Institute; The Scripps Research Institute

## Abstract

The consumption of alcohol and caffeine affects the lives of billions of individuals worldwide. Although recent evidence indicates that caffeine impairs the reinforcing properties of alcohol, a characterization of its effects on alcohol-stimulated mesolimbic dopamine (DA) function was lacking. Acting as the pro-drug of salsolinol, alcohol excites DA neurons in the posterior ventral tegmental area (pVTA) and increases DA release in the nucleus accumbens shell (AcbSh). Here we show that caffeine, via antagonistic activity on A2A adenosine receptors (A2AR), prevents alcohol-dependent activation of mesolimbic DA function as assessed, in-vivo, by brain microdialysis of AcbSh DA and, in-vitro, by electrophysiological recordings of pVTA DA neuronal firing. Accordingly, while the A1R antagonist DPCPX fails to prevent the effects of alcohol on DA function, both caffeine and the A2AR antagonist SCH 58261 prevent alcohol-dependent pVTA generation of salsolinol and increase in AcbSh DA in-vivo, as well as alcohol-dependent excitation of pVTA DA neurons in-vitro. However, caffeine also prevents direct salsolinol- and morphine-stimulated DA function, suggesting that it can exert these inhibitory effects also independently from affecting alcohol-induced salsolinol formation or bioavailability. Finally, untargeted metabolomics of the pVTA showcases that caffeine antagonizes alcohol-mediated effects on molecules (e.g. phosphatidylcholines, fatty amides, carnitines) involved in lipid signaling and energy metabolism, which could represent an additional salsolinol-independent mechanism of caffeine in impairing alcohol-mediated stimulation of mesolimbic DA transmission. In conclusion, the outcomes of this study strengthen the potential of caffeine, as well as of A2AR antagonists, for future development of preventive/therapeutic strategies for alcohol use disorder.

## Introduction

Caffeine and ethyl alcohol (alcohol) are the two most consumed psychopharmacologically active substances in the world (1, 2). The pharmacological consequences of their combined use have been extensively investigated, but different studies have produced conflicting data that diverge based on species, strains, dosages, routes, and schedules of administration. In this fragmented scenario, it is difficult to capture a unique pattern of the influence of caffeine on alcohol effects but, as far as the effects on alcohol consumption are concerned, it seems that caffeine may exert bi-directional influences depending on several experimental parameters (3–9).

To further characterize caffeine-alcohol interaction, we focused our research on the potential ability of caffeine to affect the neurophysiological and neurochemical processes underlying the reinforcing properties of alcohol. In particular, we previously showcased that caffeine, at doses borderline for eliciting arousal (10, 11) and locomotor activity (7, 12), can functionally antagonize alcohol reinforcement by demonstrating that it could prevent the acquisition of alcohol-elicited conditioned place preference and aversion (13), pointing to the dopamine (DA)-dependent underlying associative learning process (14) as the possible mechanism targeted by caffeine to gain this behavioral outcome. This hypothesis was grounded in the observations that alcohol-elicited place conditioning is prevented by DA receptor antagonists (15, 16), that caffeine exerts, through an antagonistic action on A_2A_ adenosine receptors (A_2A_R) (17), a direct negative control on neuronal firing of DA cells in the posterior ventral tegmental area (pVTA) (18), and that caffeine administration prior to alcohol also prevents its DA-dependent (19) ability to increase the expression of phosphorylated Extracellular signal Regulated Kinase (pERK) in the shell of the nucleus accumbens (AcbSh) (13, 20). Notably, increased pERK expression is a DA receptor-dependent marker of activation of mesolimbic DA transmission by alcohol (19, 21) and other addictive substances (22, 23), but not by caffeine (22, 24), as well as a DA-dependent mechanism at the basis of associative learning (14, 25–27).

The mechanism by which alcohol activates mesolimbic DA transmission has been the subject of intense research for decades, as this pathway is critical in mediating the reinforcing effects of alcohol, as well as other drugs of abuse (28, 29). Even in humans, positron emission tomography studies have shown that alcohol induces a release of DA in the ventral striatum (30), and that this fast release of DA is associated with alcohol-induced reinforcing effects and acquisition of conditioned responses (31). In this regard, the metabolic conversion of alcohol into acetaldehyde has been recognized as critically involved (32, 33), and this suggestion was further extended by the observation that another molecule, 1-methyl-6,7-dihydroxy-1,2,3,4-tetrahydroisoquinoline (salsolinol), obtainable by *Pictet-Spengler* condensation of acetaldehyde and DA, could be responsible for the reinforcing properties of alcohol and for its addictive potential (34, 35). This hypothesis was recently substantiated by multiple robust lines of evidence. The first line of evidence refers to experiments showing that systemic (36) or local (37) salsolinol administration elicits conditioned place preference, exerts alcohol-like motivational/sensitization effects (38, 39) and leads to excessive alcohol intake (39). The second line of evidence refers to an *in-vitro* electrophysiological study, in which the ability of alcohol to stimulate the firing rate of DA neurons of the pVTA critically depended on the availability of DA, as well as on the metabolic conversion of alcohol into acetaldehyde (40). Finally, direct evidence was provided also by Bassareo et al. (2021), in which the systemic administration of alcohol resulted in the *in-vivo* formation of salsolinol in the pVTA which was connected, in a mechanistic- and time-locked manner, to increased DA transmission in the AcbSh *via* m opioid receptor (41). In addition, this study also demonstrated that the inhibition of brain catalase, the enzyme responsible for alcohol oxidation into acetaldehyde, prevents both the formation of salsolinol in the pVTA and the increase of DA transmission in the AcbSh (41).

Hence, in order to understand the mechanistic influence of caffeine on DA-mediated alcohol effects (13, 20), we verified whether caffeine can affect the ability of alcohol, administered at a dose that results in mild behavioral activation (42–44), to activate DA transmission in the AcbSh, and if this influence also involves the alcohol-dependent generation and availability of salsolinol in the pVTA (41). Moreover, since caffeine is an A_1_R and A_2A_R antagonist (45), we also verified if the effects of caffeine could be attributable to an action onto adenosine receptors. To this end, the effects of caffeine, and of the selective A_1_R and A_2A_R antagonists, DPCPX and SCH 58261, on alcohol-stimulated DA transmission in the AcbSh and newly formed salsolinol in the pVTA (41) were simultaneously investigated through *in-vivo* dual probe brain microdialysis. Additionally, a catalase-mediated synthesis of salsolinol was set up, *in-vitro*, to verify whether caffeine, similarly to the non-competitive catalase inhibitor 3-amino-1,2,4-triazole (3AT), could prevent the formation of salsolinol by directly inhibiting the enzyme. Moreover, to further characterize the mechanism of action of caffeine on alcohol-mediated stimulation of mesolimbic DA neurons, *in-vitro* patch-clamp recordings from pVTA slices were performed. Additionally, to verify whether caffeine could also show effects unrelated to salsolinol generation, we verified its activity on the enhancement of mesolimbic DA transmission mediated by exogenous salsolinol, as well as by another m receptor agonist, morphine. Finally, we also performed region specific untargeted metabolomics of the pVTA in alcohol-treated rats, with and without caffeine pre-treatment, to detect changes in the biochemical profiles that might also be related to the stimulatory effects of alcohol on mesolimbic DA function.

## Materials And Methods

### Animals

Male Sprague Dawley rats (275–325 g) (Charles River, Calco, Italy; San Diego, California, US) had access to water and food *ad libitum* and were handled in accordance with the guidelines for care and use of experimental animals of the European Community Council (2010/63/UE L 276 20/10/2010), with Italian law (DL 04.03.2014, N° 26; Authorization n° 371/2020-PR), with the National Institutes of Health Guide for the Care and Use of Laboratory Animals and the Institutional Animal Care and Use Committee of The Scripps Research Institute. Every effort was made to minimize suffering and reduce the number of animals used.

### Drugs

Alcohol 1 g/kg (5.8 mL/kg) (Sigma-Aldrich, Milan, Italy) 20% (v/v) in water was administered intragastrically (i.g.). Caffeine (3 and 15 mg/kg) (Sigma-Aldrich, Milan, Italy) was dissolved in saline (3 mL/kg) and administered intraperitoneally (i.p.) 20 min before water or alcohol or dissolved in normal Ringer (see below) to 10 μM to be delivered by reverse dialysis in the pVTA, starting 30 min before water or alcohol. DPCPX and SCH 58261 (Tocris, Bristol, UK) were suspended in saline with 0.3% Tween-80 and in 0.5% methyl cellulose, respectively. Both drugs were administered i.p., at the dose of 2 mg/kg, 20 min before water or alcohol. (±)-Salsolinol (Santa Cruz Biotechnology Inc., Dallas, TX, United States) was dissolved in normal Ringer (see below) to 10 nM and delivered by reverse dialysis in the pVTA. The doses and the concentrations of alcohol (40, 41, 46–49), caffeine (10, 13, 24), DPCPX (50), SCH 58261 (51), salsolinol (37, 40, 52) and morphine (40) were selected based on previous literature.

### Microdialysis experiments

Vertical probes were stereotaxically implanted in the pVTA and AcbSh according to the rat brain atlas of Paxinos and Watson (1998) (53): AP: −5.8 mm and ML: ±0.5 mm from bregma and DV: −8.0 mm from dura, for the pVTA; AP: 1.8 mm and ML: ±1 mm from bregma and DV: −7.6 mm from dura, for the AcbSh. Probes were implanted ipsilaterally, at random distribution between left and right brain sides. The location of the probes was reconstructed and referred to the rat brain atlas plates (53) through histological analysis. On the experiment day, pVTA and AcbSh probes were connected to an infusion pump and perfused with normal Ringer (in mM: 147 NaCl, 4 KCl, 2.2 CaCl_2_) at flow-rate of 1 μl/min. Dialysate samples (10 μL) were injected without purification into a high-performance liquid chromatograph (HPLC) to simultaneously quantify salsolinol and DA as previously described (41). Sensitivity of the assay was 5 femtomoles/sample.

### Electrophysiological experiments

Rat brain slices were prepared as previously described (54). In brief, animals were decapitated under 5% isoflurane anesthesia. Brains were harvested and transferred to a modified ice-cold artificial cerebrospinal fluid (aCSF) solution containing (in mM): 220 sucrose, 2 KCl, 0.2 CaCl_2_, 6 MgSO_4_, 26 NaHCO_3_, 1.3 NaH_2_PO_4_, and 10 D-glucose (pH 7.4, adjusted by aeration with 95% O_2_ and 5% CO_2_). Horizontal brain slices containing the pVTA were sectioned (260 μm) in ice-cold modified aCSF using a Leica VT1200S vibratome (Leica, Heidelberg, Germany). Slices were transferred to a nylon mesh immersed in standard aCSF containing (in mM): 126 NaCl, 3 KCl, 2 CaCl_2_, 1 MgCl_2_, 26 NaHCO_3_, 1.25 NaH_2_PO_4_, and 10 D-glucose (pH 7.4, adjusted by aeration with 95% O_2_ and 5% CO_2_). After incubation for at least 40 min at 35°C, followed by at least 1 h at room temperature, the hemi-slices were transferred to the recording chamber and continuously perfused with standard aCSF at a flow rate of ~2 mL/min. The bath temperature was maintained at 33°C for all recordings.

Patch-clamp recordings from pVTA dopaminergic neurons were performed as previously described (54). Recording pipettes were prepared from borosilicate capillaries with an internal filament using a P-97 Flaming Brown micropipette puller (Sutter Instruments, Novato, CA, USA). The resistance of the pipettes ranged from 4.5 to 6.0 MΩ when they were filled with the following solution (in mM): 135 potassium gluconate, 10 MgCl_2_, 0.1 CaCl_2_, 1 EGTA, 10 Hepes-KOH (pH 7.3), and 2 ATP (disodium salt). Signals were recorded with an Axopatch 200-B amplifier (Axon Instruments Inc., San Jose, CA, USA), filtered at 2 kHz, and digitized at 5 kHz. The pClamp 9.2 software (Molecular Devices, Union City, CA, USA) was used to measure and analyze the firing rate and other membrane kinetic parameters of pVTA neurons and the occurrence of HCN-mediated /_h_ currents (see below). The cell-attached configuration was used to monitor the spontaneous and pharmacologically conditioned firing rate of DA neurons. After obtaining a pipette-membrane seal with a GΩ resistance, at least 10 min were allowed before recording to obtain a stable and regular spontaneous firing rate. In addition, the whole-cell configuration was obtained at the end of each recording to determine the presence of /_h_ currents, to confirm the identity of pVTA DA neurons (55). Accordingly, in our experimental conditions, identified pVTA DA neurons showed both a robust /_h_ (mean amplitude: −134.4 ± 15 pA, n = 60) in response to a single hyperpolarizing voltage step, from −65 to −115 mV, and a spontaneous regular firing rate of action potentials (4.26 ± 1.3 Hz n = 30). In each recording, after 3 min of recording a stable basal firing rate, different drugs were perfused: 60 mM alcohol (5 min), 10 μM caffeine (10 min), 10 nM salsolinol (10 min), 10 μM SCH 58261 (10 min), 10 mM DPCPX (10 min), and 1 μM morphine (10 min).

### *In-vitro* synthesis of salsolinol

The protocol followed to synthesize salsolinol was an adaptation of Akbayeva et al., 2023 (56) to obtain a catalase-mediated oxidation of alcohol into acetaldehyde and the production of salsolinol in presence of DA *via Pictet-Spenlger* reaction. The blank consisted of bovine catalase (Sigma Aldrich, Italy) at 0.33 mg/mL (666.67–1666.67 units/mL) and DA hydrochloride (Sigma Aldrich, CAS No. 62-31-7) at 1.5 mM dissolved in PBS. Triplicates of blank, blank + 0.05 M catalase inhibitor 3AT (Sigma Aldrich, Italy), and blank + 0.05 M caffeine (Sigma Aldrich, Italy) were kept in an agitator at 37°C for 20 min. After that, PBS or 1 mM alcohol in PBS + 0.06 M hydrogen peroxide (Sigma Aldrich, Italy) in PBS were added to the solutions and the samples were placed back in an agitator at 37°C for additional 30 min. The reactions were then quenched with formic acid (FA, final concentration 1% v/v). The same steps were also followed using a more diluted catalase solution (0.0033 mg/mL or 6.67–16.67 units/mL). Samples were centrifuged at 4 °C for 15 min at 14 000 g, and the supernatant was collected and diluted 1:1000 in LC graded H_2_O before untargeted metabolomics analysis.

### pVTA harvesting and sample preparation

Rats were randomly divided into 4 experimental groups: saline-water, saline-alcohol, caffeine-water, caffeine-alcohol. Subjects received pre-treatment with saline or caffeine i.p. (15 mg/kg). Twenty minutes after pre-treatment, rats were treated with water or alcohol (1 g/kg) i.g. and returned to their home cages. After 30 min from alcohol treatment, rats were decapitated under 5% isoflurane deep anesthesia, brains were removed and pVTA from both hemispheres harvested, weighted, and immediately frozen in dry ice. Pre-chilled LC graded 50% MeOH:H_2_O containing 1 μM sulfadimethoxine, as an internal standard, was added to each pVTA sample to obtain a final 1:20 w/v ratio. One 5 mm stainless steel bead was added to each sample before homogenization at 25 Hz for 5 min (TissueLyser II, Qiagen). Samples were left to incubate for 1 h at 4 °C before centrifugation at 14 000 g for 15 min at 4 °C. In separate Eppendorf’s tubes, 900 μL of supernatant was collected and added to 180 μL of formic acid (500 nM). Samples were then centrifuged again for 10 min at 14 000 g and 4 °C. The collected supernatant (1 mL) was then dried overnight in a speed vacuum concentrator. Samples were stored at −80°C and on the day of the untargeted metabolomics experiments were reconstituted in 200 μL of 50% acetonitrile (ACN) and vortexed.

### Untargeted metabolomics and *in-vitro* synthesis of salsolinol experiments

For the metabolomics experiments, a Vanquish ultra-high performance liquid chromatography (UHPLC) system coupled to a Q Exactive quadrupole orbitrap mass spectrometer (Thermo Fisher Scientific, Waltham, MA, USA) was used. Samples (5 μL) were injected into a Kinetex C18 column (50 × 2.1 mm, 1.7 μM particle size, 100 A pore size; Phenomenex, Cat#00B-4475-AN) at 30 °C column temperature. A flow rate of 0.5 mL/min was used for both the *in-vitro* synthesis of salsolinol and pVTA experiments with elution carried out using LC grade H_2_O (A) and 100% ACN (B), both acidified with 0.1% FA. Different elution gradients were used. For the *in-vitro* synthesis of salsolinol experiment: 0–1 min 0.1 % B, 1–3 min 0.1–40 % B, 3–3.5 min 40–100 % B, 3.5–5 min 100% B, 5–5.1 min 100–0.1% B, 5.1–6.5 min 0.1 % B; for the pVTA experiment: 0–1 min 5 % B, 1–7 min 5–100 % B, 7–7.5 min 100 % B, 7.5–8 min 100–5% B, 8–10 min 5 % B.

The mass spectrometer was operated in data-dependent acquisition (DDA) mode, and it was used in an *m/z* range from 100 to 1500 Da in the pVTA experiments and 50 to 750 Da in the *in-vitro* synthesis of salsolinol experiment, operating in positive ionization mode. Full scan MS1 was performed at 1e6 with a resolution of 35 000 and 70 000 for the pVTA and *in-vitro* synthesis of salsolinol experiment respectively, with a maximum ion injection time (IT) of 100 ms. MS2 experiments were performed at a resolution of 17 500 with maximum IT of 100 ms for pVTA and 50 ms for catalase, and TopN was used for the 5 most abundant precursor ions per MS2. The MS2 precursor isolation window was set to 1 *m/z* with no offset. The step collision energy was set to 20 eV, 30 eV, and 40 eV.

### Metabolomics data processing

Acquired .raw files were converted into open-access .mzML format using MSConvert 3.0.23 (57). Both .raw and .mzML files have been deposited and can be downloaded from public metabolomics repository GNPS/MassIVE (https://massive.ucsd.edu/) under the accession codes MSV000094216 (pVTA experiment) and MSV000094218 (*in-vitro* synthesis of salsolinol experiment). Feature detection and extraction was performed using MZmine 3.9 (58). Briefly, mass detection noise for MS1 and MS2 was set at 5e4 and 1e3 respectively. ADAP chromatogram builder parameters were set as 4 minimum consecutive scans, 8e4 minimum absolute height, and 10 ppm *m/z* tolerance. Local minimum feature resolver module was set at 85% chromatographic threshold, 0.05 minimum search range RT, and 1.70 minimum ratio of peak top/edge. The 13C isotope filter was applied with an *m/z* tolerance of 5 ppm and a retention time tolerance of 0.03 minutes. Features were aligned using a *m/z* tolerance of 5 ppm and retention time tolerance of 0.2 minutes, with weight for *m/z* over RT was set to 3:1. Features not present in at least two samples and without MS2 acquisition were discarded. Finally, a feature list and two .mgf files, one for molecular networking (59) and one for SIRIUS (60), were exported for downstream analysis.

### Metabolomics data analysis

Feature-based molecular networking analyses (61) were performed on GNPS (https://gnps.ucsd.edu/) and can be accessed for both pVTA (https://gnps.ucsd.edu/ProteoSAFe/status.jsp?task=abb23428a158496b8bd0c689a43d2940) and catalase (https://gnps.ucsd.edu/ProteoSAFe/status.jsp?task=60b61623c3874081a9b263371b03d49a) experiments. Briefly, tolerances for both precursor ion and fragment ions were set at 0.02 Da. For networking, a minimum modified cosine score of 0.7 and minimum number of matching peaks of 3 were set. Same parameters were set for library search. Generated annotation table was used for subsequent analysis and network were visualized using Cytoscape 3.10 (62). Compound classes were predicted using CANOPUS (63) in SIRIUS 5.8.5. For the *in-vitro* synthesis of salsolinol experiment, a targeted peak extraction was also performed using Skyline v23.1 (64). Feature list was imported in R 4.2.2 (The R Foundation for Statistical Computing, Vienna, Austria) for univariate and multivariate analyses. Feature list was first cleaned though blank filtering, only features with peak area ratio > 5 compared to blanks were kept. Data quality was assessed calculating coefficient of variance of internal standard in the samples and of the 6 standards present in the quality control samples (QCmix). Principal component analysis (PCA), via *mixOmics* v 6.22 package, was used to inspect data and visualize possible outliers. Before ordination, data was robust center log ratio transformed using *vegan* v 2.6. Batch effects were corrected using the remove Batch Effect function of lim *ma* v 3.54. Supervised multivariate partial least square discriminant analysis (PLS-DA) models were generated using *mixOmics* and performance (classification error rate) was assessed using a 4-folds cross validation.

### Statistical analysis

Statistical analysis was carried out either via Statistica 8.0 (StatsSoft Inc., Tulsa, OK, USA) or PRISM, GraphPad 8 Software (San Diego, CA, USA) with significance set for all the experiments at p < 0.05. For microdialysis experiments, basal dialysate salsolinol and DA were calculated as the average ± SEM of the last three consecutive samples differing by no more than 10%, collected during the time preceding each treatment. Changes in dialysate salsolinol and DA were expressed as fmol/10 μl of dialysate and were analyzed by two- or three-way Analysis of Variance (ANOVA) with repeated measures over time. For electrophysiology experiments, all data are reported as mean ± SEM. Before ANOVA analyses, the normal distribution of data was evaluated by skewness and kurtosis, and homoscedasticity via the Bartlett test. Comparisons among experimental conditions were obtained using at least n=4 rats/group and was performed by one-way ANOVA followed by Tukey’s post-hoc test. Detailed statistical analysis for microdialysis and electrophysiology experiments is available in ***Supplementary Tables 1 and 2***.

## Results

### Effects of caffeine and adenosine receptor antagonists on alcohol-elicited pVTA salsolinol formation and AcbSh DA increase *in-vivo*.

**Figure 1:**
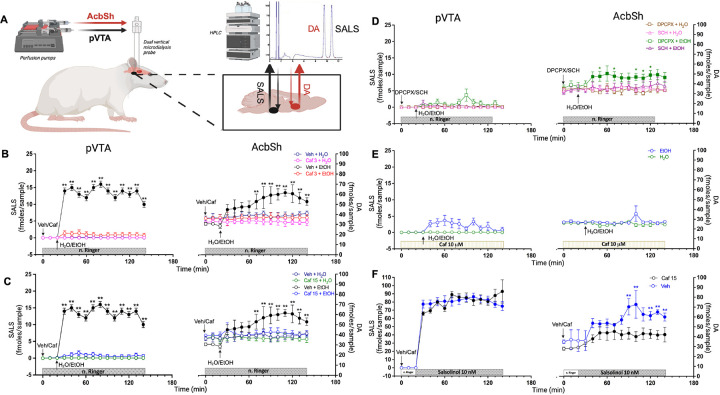
Effects of caffeine, DPCPX, and SCH 58261 on alcohol-induced pVTA salsolinol formation and AcbSh DA increase, and effects of caffeine on salsolinol bioavailability and AcbSh DA increase during pVTA perfusion with salsolinol. **(A)** Schematic representation of dual probe *in-vivo* brain microdialysis procedures, sampled areas and neurotransmitters recorded. (**B-F**) Effects of i.p. administration of Caf (3 mg/kg) (**B**), Caf (15 mg/kg) (**C**), DPCPX or SCH (**D**) and of pVTA perfusion with Caf (**E**) on pVTA SALS formation and AcbSh DA enhancement induced by i.g. EtOH, and (**F**) effects of i.p. administration of Caf (15 mg/kg) on pVTA SALS concentration and ipsilateral AcbSh DA transmission during pVTA perfusion with SALS. Horizontal bars depict the duration and content of the pVTA perfusion along the experiments. Vertical arrows indicate the last pVTA or AcbSh microdialysis sample before Veh, Caf, DPCPX or SCH and water or EtOH administrations. Filled symbols indicate samples representing p<0.001 vs. basal; **p<0.01 vs Caf (3 mg/kg) + EtOH, vs Caf (15 mg/kg) + EtOH, and vs Caf (15 mg/kg) + SALS; *p<0.05 vs DPCPX+H_2_O. Veh-H_2_O (n=4); Veh-EtOH (n=6); Caf (3 mg/kg)-H_2_O (n=4); Caf (15 mg/kg)-H_2_O (n=4); Caf (3 mg/kg)-EtOH (n=11); Caf (15 mg/kg)-EtOH (n=12); DPCPX-H_2_O (n=3); SCH- H_2_O (n=3); DPCPX-EtOH (n=5); SCH-EtOH (n=6); Caf (10mM)-H_2_O (n=3); Caf (10mM)-EtOH (n=8); Veh-SALS (n=3); Caf (15mg/kg)-SALS (n=5). Abbreviations: Veh: Saline; Caf: Caffeine; H_2_O: Water; EtOH: Alcohol; SCH: SCH 58261; SALS: Salsolinol.

Simultaneous dual probe *in-vivo* brain microdialysis was used to verify the effects of caffeine and adenosine receptors antagonists on alcohol-dependent salsolinol generation, in the pVTA, and DA transmission, in the AcbSh (Figure 1A). Alcohol elicited the formation of salsolinol in the pVTA and stimulated DA transmission in the AcbSh and caffeine significantly prevented these effects (Figure 1B ***and*** 1C, Three-way ANOVA followed by Tukey’s post hoc test). No production of salsolinol was observed after alcohol administration also when DPCPX or SCH 58261 were used as pre-treatment (Figure 1D, Three-way ANOVA, p > 0.05). In addition, caffeine and SCH 58261, but not DPCPX, prevented the increase of DA after alcohol administration (Figure 1B–D, Three-way ANOVA followed by Tukey’s post hoc test). *In-vivo* brain microdialysis was also used to verify the effect of intra-pVTA caffeine on alcohol-dependent salsolinol generation in the pVTA and DA transmission in the AcbSh. Salsolinol and DA concentrations during reverse dialysis application of caffeine in the pVTA failed to reveal any effect of alcohol (Figure 1E, Two-way ANOVA p > 0.05). Given that local application of caffeine by reverse dialysis prevented both systemic alcohol-dependent salsolinol formation in the pVTA and DA increase in the AcbSh, these results suggest that the systemic effects of caffeine might be mediated by a direct action on the pVTA.

Additionally, an *in-vitro* synthesis of salsolinol was set up to verify whether caffeine could prevent salsolinol formation acting as a catalase inhibitor. Specifically, the ability of the catalase-inhibitor 3AT and caffeine to prevent catalase-mediated alcohol oxidation to acetaldehyde and, consequently, its condensation with DA to generate salsolinol were investigated (***Supplementary Figure 1A, B***). As expected, salsolinol formation was catalase-dependent, as lowering the units/mL of the enzyme also reduced salsolinol abundance (***Supplementary Figure 1A***, One-way ANOVA followed by Tukey’s post hoc test). Caffeine, differently from 3AT, did not prevent the formation of salsolinol (***Supplementary Figure 1A***, One-way ANOVA followed by Tukey’s post hoc test), ruling out the possibility of a direct inhibitory activity on catalase.

*In-vivo* brain microdialysis was also used to verify the effects of caffeine on salsolinol bioavailability in the pVTA and on salsolinol-induced DA transmission in the AcbSh. The systemic administration of caffeine failed to significantly affect pVTA salsolinol concentrations during pVTA perfusion with exogenous salsolinol (Figure 1F, Two-way ANOVA p > 0.05), pointing out that caffeine does not affect salsolinol bioavailability. However, caffeine pre-treatment significantly reduced the increase of AcbSh DA induced by reverse dialysis of exogenous salsolinol in the ipsilateral pVTA (Figure 1F, Two-way ANOVA followed by Tukey’s post hoc test). These last results suggest that other mechanisms of action in the pVTA, in addition to the prevention of alcohol-induced salsolinol formation, should be envisioned to explain caffeine inhibitory effects on alcohol-induced increase of mesolimbic DA transmission.

### Effects of caffeine on the excitation of pVTA DA neurons induced by alcohol, morphine, and salsolinol: *in-vitro* electrophysiological experiments

**Figure 2:**
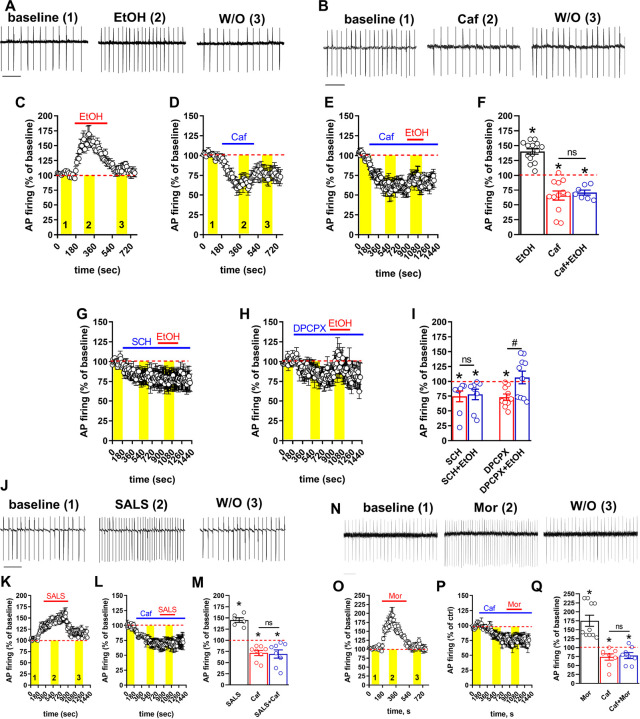
Effects of alcohol, caffeine, salsolinol, and morphine on the firing rate of rat pVTA DA neurons. **(A, B)** Representative traces of spontaneous firing recorded from single DA neurons before (1, baseline), during (2), and after (3, washout) bath application of 60 mM EtOH (**A**), 10 μM Caf (**B**), of 10 nM SALS (**J**), and of 1 μM Mor (**N**). Scale bar: 1 s. (**C-E,G-H, K-L, O-P**) Graphs showing the effects of EtOH (**C**), Caf (**D**) and their combination (**E**), of the combination of 10 μM SCH (**G**) or 10 μM DPCPX (**H**) with 60 mM EtOH, of 10 nM SALS alone (**K**) and in association with 10 μM Caf (**L**), and of 1 μM Mor alone (**O**) and in association with 10 μM Caf (**P**) on the firing rate of DA neurons. Data are expressed as mean ± SEM. (**F,I, M, Q**) The bar graphs summarize the percentage of change from baseline produced by EtOH and Caf alone and by their combination (n=33 neurons from 11 rats) (**F**), the effects of SCH and DPCPX on the stimulatory effect of EtOH (n = 36 neurons from 18 animals) (**I**), the effects of SALS and Caf when bath perfused alone or during their association (n = 22 neurons from 11 rats) (**M**), the effects of Mor alone and in combination with Caf n = 22 neurons from 11 rats (**Q**). Data are derived from the graphs **C-E,G-H, K-L, O-P** (average highlighted by the yellow bars). One-way ANOVA, * p < 0.05 versus baseline; #p < 0.05 versus DPCPX alone. Abbreviations: EtOH: Alcohol; Caf: Caffeine; SCH: SCH58261; SALS: Salsolinol; Mor: Morphine.

To further characterize the effects of caffeine on alcohol-induced stimulation of mesolimbic DA signaling, *in-vitro* patch-clamp recordings from pVTA slices were performed. As expected from previous reports (47–49, 65), acute perfusion of 60 mM alcohol significantly increased (40.1 ± 4.4 %) the firing rate of pVTA DA neurons, an effect that was promptly reversed by drug washout (Figure 2A, B, F, one-way ANOVA followed by Tukey’s post hoc test). In contrast, 5 min of acute perfusion with 10 μM caffeine significantly decreased (−34.3 ± 7.7 %) DA neuron firing rate, (Figure 2C, D, F, one-way ANOVA followed by Tukey’s post hoc test). The modulatory effect of alcohol on the firing rate of pVTA DA neurons was completely suppressed in the presence of 10 μM caffeine (Figure 2E, F, one-way ANOVA followed by Tukey’s post hoc test).

*In-vitro* patch-clamp recordings from pVTA slices were also performed to test whether this effect of caffeine was mediated by an antagonistic component on A_1_R or A_2A_R. Independent bath perfusion with either antagonist decreased the firing rate of pVTA DA neurons (Figure 2G–I, one-way ANOVA followed by Tukey’s post hoc test). Interestingly, while the effect of alcohol was completely blocked by the A_2A_R antagonist SCH 58261 (Figure 2G, I, one-way ANOVA followed by Tukey’s post hoc test), it was indistinguishable from its effect when tested alone in presence of DPCPX (Figure 2H, I, one-way ANOVA followed by Tukey’s post hoc test), suggesting that the ability of caffeine to suppress the modulatory effect of alcohol on DA firing rate is mediated by an action on A_2A_R, but not A_1_R.

Finally, *in-vitro* patch-clamp recordings were performed to investigate whether caffeine could interfere with the positive modulatory effect of salsolinol or morphine on DA neuron firing rate. Accordingly, the acute perfusion of 10 nM salsolinol significantly increased (45.1 ± 5.2 %) the firing rate in pVTA DA neurons, an effect that was reversed 5 min after of drug removal (Figure 2J, K, M, one-way ANOVA followed by Tukey’s post hoc test). The effect of salsolinol on DA neuron firing rate was completely suppressed in the presence of caffeine (Figure 2L, M, one-way ANOVA followed by Tukey’s post hoc test), reinforcing the results obtained *in-vivo* (Figure 1F). Similarly, the acute perfusion of 1 μM morphine caused a strong increase (75.1 ± 15.2 %) in firing rate in pVTA DA neurons, which was easily washed out after 5 min after of drug removal (Figure 2N, O, Q, one-way ANOVA followed by Tukey’s post hoc test). The modulatory effect of morphine was also completely abolished in the presence of caffeine (Figure 2P, Q, one-way ANOVA followed by Tukey’s post hoc test). These last results confirm that, in addition to the prevention of alcohol-induced salsolinol formation, other mechanisms must be involved in caffeine’s inhibitory effects on alcohol-induced increase of mesolimbic DA transmission.

### Effects of caffeine on the pVTA biochemical profiles in alcohol-treated rats

**Figure 3:**
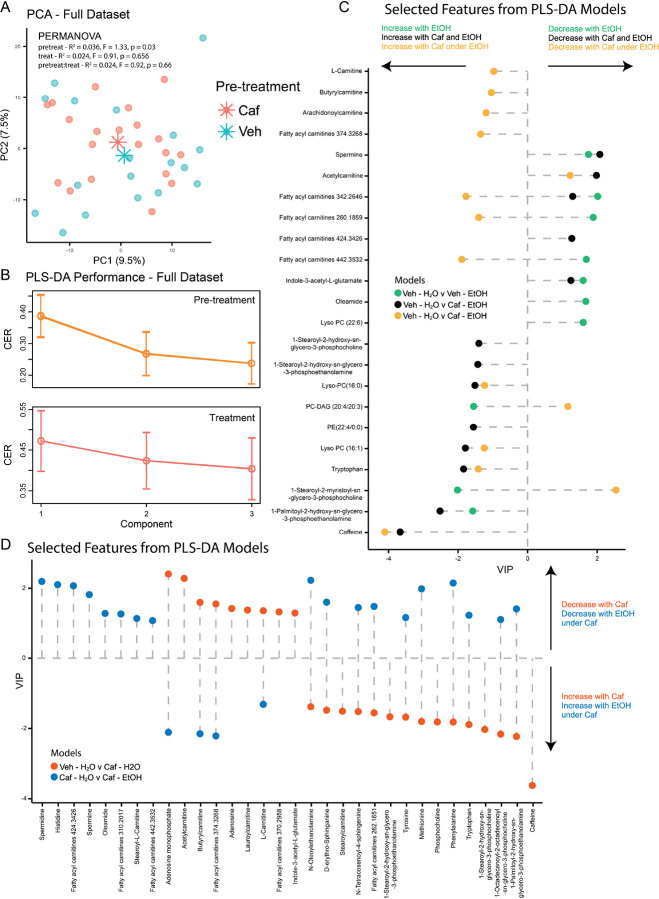
Effects of caffeine and alcohol on the biochemical profiles of rats pVTA. (**A**) Unsupervised PCA of complete dataset highlighted pre-treatment effect on pVTA biochemical profiles (PERMANOVA, R^2^ = 0.036 and p = 0.03). **B**) Supervised PLS-DA models of complete dataset showed a stronger effect of pre-treatment over treatment. Classification error rate (CER) calculated with 5-fold cross validation and 999 permutations. VIP scores of pairwise PLS-DA models Saline-Water v Saline-Alcohol, Saline-Water v Caffeine-Alcohol and Saline-Alcohol v Caffeine-Alcohol (**C**) and Saline-Water v Caffeine-Water and Caffeine-Water v Caffeine-Alcohol (**D**) are plotted for molecules of interest. Stratified models performance and features with VIP > 1 are listed in Supplementary Tables 3–6. N=9 per group. Abbreviations: Veh: Saline; Caf: Caffeine; H_2_O: Water; EtOH: Alcohol.

In order to detect additional mechanisms of action of caffeine independent from salsolinol formation and bioavailability, untargeted metabolomics analysis of rats pVTA was used to assess potential changes in the biochemical profiles in response to alcohol or water treatment, with or without caffeine pre-treatment. Unsupervised PCA and supervised PLS-DA of all the analyzed samples revealed a stronger effect of pre-treatment over treatment (Figure 3A, B, PERMANOVA pre-treatment R^2^ = 0.03 and p = 0.03; PLS-DA pre-treatment CER = 0.23; PLS-DA treatment CER = 0.40). More specifically, alcohol administration moderately affected pVTA biochemical profiles, pairwise PLS-DA model saline-water vs saline-alcohol (CER = 0.38), mostly influencing molecules involved in lipid signaling and energy metabolism, such as phosphatidylcholines (PCs), lyso-PCs, fatty amides, and carnitines. Alcohol increased the abundance of stearoyl-myristoyl-glycero-phosphocholine, PC-DAG, and palmitoyl-hydroxy-glycero-phosphoethanolamine, and decreased Lyso-PC (22:6), oleamide, spermine, indole-acetyl-glutamate and three predicted acyl-carnitines (Figure 3C, ***Supplementary Table 3***). Interestingly, combined administration of both alcohol and caffeine, pairwise PLS-DA model saline-water vs caffeine-alcohol (CER = 0.27), did not highlight differences in stearoyl-myristoyl-glycero-phosphocholine, PC-DAG, Lyso-PC (22:6), oleamide and in two of the predicted acyl-carnitines, suggesting that caffeine prevented alcohol-induced specific alterations of these molecules (Figure 3C). Pairwise PLS-DA model saline-water vs caffeine-alcohol (CER = 0.27) also highlighted an increase of indole amino acids, phenylalanine, tryptophan, tyrosine, arginine, methionine, PCs (hexadecanoyl-, hexadecyl-, octadecanoyl-, stearoyl-hydroxy- glycero-phosphocholine) and phosphatidylethanolamines (PEs) (palmitoyl-hydroxy-glycero-phosphoethanolamine and stearoyl-hydroxy-glycero-phosphoethanolamine) and a decrease in spermine and spermidine, oleoylethanolamine, arachidonoylthio-PC, adenosine, and acetyl-carnitine (***Supplementary Table 4***).

Pre-treatment with caffeine had the biggest impact on the pVTA biochemical profiles, pairwise PLS-DA model saline-water vs caffeine-water (CER = 0.18). Caffeine increased the abundance of several amino acids, such as the indole amino acids, phenylalanine, tryptophan, and tyrosine, methionine, arginine, and gamma-glutamylglutamate, glycerophospholipids, including different PCs (heptadecanoyl-, hexadecyl-, octadecanoyl-, stearoyl-hydroxy-glycero-phosphocholine) and PEs, such as palmitoyl-hydroxy-glycero-phosphoethanolamine and stearoyl-hydroxy-glycero-phosphoethanolamine, sphingolipids, like tetracosenoyl-sphingenine and erythro-sphinganine, several fatty amides, including oleoylethanolamine and predicted ones, inosine, and 6-oxopurine. On the contrary, the abundance of adenosine and adenosine monophosphate, of indole-acetyl-glutamate, and of arachidonoylthio-PC appeared to decrease in response to caffeine. Moreover, caffeine generally reduced the carnitine pool: accordingly, L-carnitine, acetyl-carnitine, butyryl-carnitine, lauroyl-carnitine and three predicted ones all decreased (Figure 3D ***and Supplementary Table 5***). However, comparison of caffeine or saline pretreatment under alcohol treatment, pairwise PLS-DA model saline-alcohol vs caffeine-alcohol (CER = 0.36) revealed that caffeine had a completely opposite effect on carnitines under alcohol treatment. Accordingly, L-carnitine, butyrylcarnitine, arachidonoylcarnitine, and other four predicted carnitines increased with caffeine pre-treatment under alcohol treatment (Figure 3C). These last results suggest that caffeine might affect carnitines pool differentially depending on the presence of alcohol.

Finally, pairwise PLS-DA model saline-alcohol vs caffeine-alcohol (CER = 0.36) also revealed that caffeine was responsible for increased abundance of indole amino acids, phenylalanine, tryptophan, and tyrosine, 6-oxopurine, niacinamide/nicotinamide and arachidonoylthio-PC, while it decreased the abundance of histidine, acetyl-arginine, acetyl-carnitine, 13-docosenamide, and some PCs, such as stearoyl-myristoyl- and octadecanoyl-glycero-phosphocholine (***Supplementary Table 6***).

## Discussion

Alcohol consumption is one of the leading risk factors for premature death and disability, contributing to approximately 2.5 million deaths each year worldwide (66). The ability of alcohol to stimulate mesolimbic DA function (67), as a requirement to exert its reinforcing effects (14, 41, 46, 65, 68–70), has critical implications for the development of alcohol use disorder (AUD) (71, 72). Recent studies have shown that alcohol excites DA neurons in the pVTA (40) and stimulates DA transmission in the AcbSh acting as the pro-drug of salsolinol (41). Caffeine is a psychopharmacological agent devoid of addictive potential (10, 14, 73, 74) and equally consumed worldwide as alcohol. The widespread diffusion of these two substances in the last decades has raised several questions on the clinical impact of their simultaneous consumption. The present study was aimed at characterizing the consequences of the interaction of a behaviorally relevant acute dose of each of these substances on DA function. The results reveal that the administration of caffeine prior to alcohol prevents its ability to generate salsolinol in the pVTA and, accordingly, to increase AcbSh DA transmission. This outcome suggests that caffeine might be preventing the ability of alcohol to increase AcbSh DA by interfering with the generation and/or with the bioavailability of salsolinol in the pVTA. However, as far as the reduction of the bioavailability is concerned, this possibility can be ruled out since salsolinol detection does not significantly differ, with and without systemic administration of caffeine, during pVTA perfusion with salsolinol. Moreover, differently from the catalase inhibitor 3AT, caffeine does not inhibit catalase-dependent formation of salsolinol *in-vitro*. Consequently, the possibility that caffeine affects salsolinol generation directly inhibiting the enzyme catalase, whose activity is necessary to salsolinol formation (40, 41), was also ruled out. Therefore, we hypothesized that caffeine could prevent alcohol stimulation on pVTA DA neurons, as well as alcohol-dependent generation of pVTA salsolinol and AcbSh DA transmission, *via* an adenosine receptor-mediated mechanism. Notably, as far as the generation of salsolinol is concerned, this was the case, since both the A_1_R and A_2A_R antagonists, DPCPX and SCH 58261, prevented the generation (and detection) of salsolinol in pVTA after alcohol administration. However, the administration of A_1_R and A_2A_R antagonists prior to alcohol revealed that these receptors differentially affect alcohol-elicited increases of AcbSh DA. Accordingly, SCH 58261, but not DPCPX, prevents the stimulation of AcbSh DA transmission by alcohol. This latter observation appears fully in agreement with the electrophysiological recordings with A_1_R and A_2A_R antagonists. Therefore, both *in-vivo* and *in-vitro* evidence indicates that the action of systemic caffeine on alcohol-stimulated AcbSh DA is mediated by the prevention of salsolinol formation in the pVTA, via A_1_R- and A_2A_R-mediated mechanism, although the finding that A_1_R blockade fails to affect alcohol-mediated increase of DA function, *in-vivo* and *in-vitro*, strongly points out a mechanism of alcohol-stimulated AcbSh DA and pVTA neuronal firing independent from salsolinol generation. Moreover, the observation that caffeine reduces the stimulation *in-vivo* (AcbSh DA, by reverse dialysis) and *in-vitro* (pVTA DA neuronal firing) by exogenous salsolinol, as well as the stimulation of pVTA DA neuronal firing *in-vitro* by morphine, confirms that salsolinol generation-independent mechanisms should be envisioned. In this regard it appears reasonable to hypothesize that under these conditions blockade of A_1_R, i.e. lack of an adenosine tone on A_1_R, frees a mechanism that, in spite of the unavailability of salsolinol in the pVTA, still results in DA neurons excitation and increased AcbSh DA release. Thus, since the local application of caffeine in the pVTA results in the same effects of the systemic one, it was reasonable to look for these additional biological mechanisms in the same region.

Accordingly, untargeted metabolomics analysis of pVTA lysates points out that both alcohol and caffeine influence the abundance of various lipids, but also that caffeine prevents alcohol-induced alterations in the concentration of most of these molecules. In addition to their structural function in cellular membranes, lipids in the brain play crucial roles in regulating various physiological processes, including signal transduction (75), synaptic plasticity (76), and the release of neurotransmitters (77). The role of lipids in addiction is well known (78, 79) and alcohol (80–82), as well as other addictive substances including morphine (83) or cocaine, can alter their signaling. Lipid signaling is involved specifically in DA mesolimbic transmission (84), by regulating reinforcing and motivational aspects of feeding (85), but also VTA DA neurons firing (86, 87). One of the lipids reduced by alcohol in the present study is oleamide. Oleamide is an endogenous fatty acid amide, derived from oleic acid, which can be synthesized in the mammalian nervous system and, among other effects, enhances the amplitude of currents gated by GABA_A_ receptors (88). Notably, we recently demonstrated that GABA_A_ agonists (89), similarly to caffeine (13), prevent alcohol- and morphine-induced conditioned place preference, as well as pERK increase in the AcbSh (90). Interestingly, recent studies revealed that intra-VTA administration of oleic acid inhibits DA tone (86), and that oleamide, acting as PPARα/CB1 receptor dual ligand, reduces alcohol intake and alcohol and oxycodone self-administration in rats (91). In the present study, caffeine prevents alcohol-induced reduction in oleamide. Moreover, caffeine also prevents alcohol-induced changes in PC and Lyso-PC which activate PPARα/γ in addition to other signaling pathways (92) , and have been suggested as potential targets for cocaine addiction (93). Interesting effects of caffeine were observed also on the carnitine pool. In fact, not only caffeine seems to prevent alcohol-induced reduction of two predicted acyl-carnitines, but it also appears to regulate carnitines abundance bidirectionally depending on the presence of alcohol. Carnitines are amino acid derivatives essential for the transportation of fatty acids into the mitochondria (94). A potentially therapeutic role of carnitines and acyl-carnitines in AUD has already been described in rodents (95, 96) and humans (97). Moreover, previous studies reported that carnitine inhibits catalase activity and prevents catalase-mediated effects of alcohol in mice (98, 99). In the present study, caffeine-induced increase in carnitine and acyl-carnitines, selectively under alcohol treatment, might have reduced catalase-mediated oxidation of alcohol, explaining the prevention of salsolinol formation in the pVTA and justifying the discrepancy between the effects of caffeine on catalase-mediated salsolinol generation *in-vivo* (preventive) and *in-vitro* (no effect).-

In conclusion, the present work reveals for the first time that caffeine prevents alcohol-induced activation of the mesolimbic DA pathway. Encouragingly, one of the few FDA-approved drugs for AUD, the m receptor antagonist naltrexone (ReVia^®^; Depade^®^), prevents the reinforcing effects of alcohol by interfering with its enhancement of the mesolimbic DA transmission (100) strengthening the potential of caffeine, and more specifically of A2_A_R antagonists, for future development of preventive/therapeutic strategies for AUD. Moreover, not only the stimulation of the mesolimbic DA pathway is the critical initiating event of the neurocircuitry of AUD, but also of addiction in general (14, 28, 29) and, since our results point out that caffeine can also prevent mesolimbic DA stimulation by the m receptor agonists, salsolinol and morphine, one of the future directions of this study will be to characterize further its effects on opioids-, as well as other drugs of abuse. More detailed studies will also be required to explain how A_2A_R antagonism elicits its inhibitory activity on alcohol stimulation as well as the differential effects of A_1_R blockade on alcohol-mediated generation of pVTA salsolinol and stimulation of AcbSh DA, and to interpret the involvement of lipid signaling in caffeine effects on alcohol activity in the mesolimbic system. One of the limitations of this study is its exclusive focus on the mesolimbic DA pathway in alcohol naïve rats, which is only representative of the initial phase of AUD. We acknowledge the role of other brain circuits in the onset and self-perpetuating cycle of AUD, as well as the importance of other stages (i.e. withdrawal, craving, relapse) of the disease. Hence, future studies are required to explore the therapeutic potential of caffeine and adenosine receptor antagonists in both naïve and dependent rats, at different stages of the disease.
